# Heterogeneous computing for epidemiological model fitting and simulation

**DOI:** 10.1186/s12859-018-2108-3

**Published:** 2018-03-16

**Authors:** Thomas Kovac, Tom Haber, Frank Van Reeth, Niel Hens

**Affiliations:** 1 Center for Statistics, I-BioStat, Hasselt University, Agoralaan building D, Diepenbeek, 3590 Belgium; 2 Expertise Centre for Digital Media, Hasselt University, Wetenschapspark 2, Diepenbeek, 3590 Belgium; 3 Centre for Health Economic Research and Modelling Infectious Diseases, Vaccine and Infectious Disease Institute, University of Antwerp, Universiteitsplein 1, Wilrijk, 2610 Belgium

**Keywords:** ODE, PDE, Infectious diseases, Epidemiology, SIR model, GPU, Asynchronous, Parallel, Particle swarm optimization, Heterogeneous computing

## Abstract

**Background:**

Over the last years, substantial effort has been put into enhancing our arsenal in fighting epidemics from both technological and theoretical perspectives with scientists from different fields teaming up for rapid assessment of potentially urgent situations. This paper focusses on the computational aspects of infectious disease models and applies commonly available graphics processing units (GPUs) for the simulation of these models. However, fully utilizing the resources of both CPUs and GPUs requires a carefully balanced heterogeneous approach.

**Results:**

The contribution of this paper is twofold. First, an efficient GPU implementation for evaluating a small-scale ODE model; here, the basic S(usceptible)-I(nfected)-R(ecovered) model, is discussed. Second, an asynchronous particle swarm optimization (PSO) implementation is proposed where batches of particles are sent asynchronously from the host (CPU) to the GPU for evaluation. The ultimate goal is to infer model parameters that enable the model to correctly describe observed data. The particles of the PSO algorithm are candidate parameters of the model; finding the right one is a matter of optimizing the likelihood function which quantifies how well the model describes the observed data. By employing a heterogeneous approach, in which both CPU and GPU are kept busy with useful work, speedups of 10 to 12 times can be achieved on a moderate machine with a high-end consumer GPU as compared to a high-end system with 32 CPU cores.

**Conclusions:**

Utilizing GPUs for parameter inference can bring considerable increases in performance using average host systems with high-end consumer GPUs. Future studies should evaluate the benefit of using newer CPU and GPU architectures as well as applying this method to more complex epidemiological scenarios.

## Background

Over the last years, substantial effort has been put into enhancing our arsenal in fighting epidemics from both technological and theoretical perspectives. For example, effective vaccines and antiviral drugs can be produced with knowledge going deep into the molecular structure of viruses, and mathematical modeling of infectious diseases helps provide insight into the disease dynamics and the design of intervention/vaccination programs. Scientists from different fields, extending from medicine and molecular biology to computer science and applied mathematics, are teaming up for rapid assessment of potentially urgent situations [1–4].

This paper focusses on the computational aspects of simulating these mathematical models and parameter inference. Infectious diseases are often modeled using ordinary and partial differential equations (ODE and PDE). However, most models are non-linear in nature and cannot be solved analytically. Therefore a numerical method is generally used to provide approximate solutions. Performing parameter inference on these models typically requires a large number of evaluations with different parameter values, which can be an incredibly computationally expensive task.

Since their inception, graphics processing units (GPUs) have been transformed from common peripherals into powerful devices that can be used for general-purpose programming [5–7]. GPUs are single-instruction multiple-data (SIMD) [8] devices. They contain chips that, in turn, contain hundreds of cores that allow hundreds of threads to be executed in parallel. CUDA [9], from NVIDIA, is a computing platform that exposes parallel compute power of NVIDIA GPUs to developers without a graphics background. However, it remains difficult to program GPUs for general-purpose use as the single-instruction, multiple-threads (SIMT) nature of GPUs does not allow all algorithms to be mapped onto a GPU.

Whereas GPUs are often exceptionally suited for solving big ODE/PDE problems (such as Computational Fluid Dynamics [10–12]), the models in infectious diseases are more troublesome; their smaller size makes it harder to optimally utilize all GPU cores and the SIMT nature introduces additional overhead which lowers performance. In contrast, simulating multiple small models simultaneously can be done efficiently on GPUs.

Inference of model parameters is usually done by maximizing the (log)likelihood such that the model accurately describes the data at hand. A number of local and global optimization methods can be used to this end. This paper focuses on particle swarm optimization (PSO): a method introduced by Kennedy et al. [13] for optimizing continuous non-linear functions. Based on the principles of bird flocking, fish schooling, and swarm theory, PSO can be implemented in a few lines of code.

Since the exchange of information between CPU and GPU is expensive [14], the common approach is to run all steps of the optimization algorithm on the GPU [15–18]. Instead, this paper proposes a heterogeneous approach for the following reasons:

- The CPU is a valuable resource that would otherwise be left idle.
- Implementing algorithms on the GPU requires thorough knowledge of hardware specifics for optimal performance.
- The sequential and branching nature of the algorithms makes them ill-suited for GPUs, since their SIMT architecture requires every thread to execute the same instruction at every moment.
- CPU implementation allows the use of scientific languages such as Python, Julia or MATLAB, significantly shortening development time.
- Several high-end implementations can be reused.

The contribution of this paper is twofold. First, an efficient GPU implementation for evaluating small-scale ODE models based on the Runge-Kutta method is presented and its scalability in number of threads and equations is investigated. Second, this paper proposes the use of an asynchronous PSO implementation, for inferring parameters of an infectious disease model, that enables efficient utilization of both CPU and GPU resources.

## Related work

GPUs have evolved to such a degree that they have outpaced CPUs in terms of processing speed. As a result, they are being used for non-graphical applications such as high-order numerical integrations [19]. Amidst a plethora of numerical integration algorithms, Runge-Kutta methods are a family of implicit and explicit iterative methods, with a wide variety of orders and schemes [20]. Seen et al. [21] implemented a Runge-Kutta-Fehlberg (commonly denoted *RK45*) with adaptive step size on an NVIDIA GPU. The authors demonstrated that the GPU outperforms a CPU implementation, given that the problem dimensions are large enough, as in 200 equations, or more.

Niemeyer et al. [22, 23] developed GPU versions of the adaptive fifth-order Runge-Kutta-Cash-Karp (RKCK) method and stabilized second-order Runge-Kutta-Chebyshev (RKC) method, which are used for problems of non-stiff and greater stiffness nature, respectively. The authors came to the same conclusion; relative simple systems of ODEs, as in 512 equations, or less, limit the number of calculations performed on the GPU, resulting in the fact that the transfer of data to and from the GPU is expensive.

Murray [24] explains that a Runge-Kutta implementation with adaptive time steps is an example of a class of problems where the task-length for individual threads is variable. In this particular case, it is possible that the step size modification and error control can differ for each thread, which results in warp divergence and therefore loss in performance. To mitigate this complication, the author suggests that multiple data items could be bundled into each thread. When a task for one item is completed, a thread may advance immediately onto the next task.

In the last decade, several studies have been conducted on PSO methods in terms of performance and efficiency, new applications, and new variants of the algorithm. Both Koh et al. [25] and Venter et al. [26] concurrently, but independently, published work on a parallel and asynchronous implementation of the PSO algorithm. Both articles propose the use of a master-slave approach, where slave processors evaluate particles and the master conducts particle updates in terms of velocity and position.

Although this paper focuses on an asynchronous CPU implementation of PSO that ships off particles to the GPU for evaluation, the work of the following authors is included for completeness, as they concentrated on implementing a GPU version of PSO. Veronese et al. [15] were the first to experiment with the PSO algorithm and CUDA. With the entire, classical, PSO algorithm ported to GPU using CUDA, they observed a significant reduction of the computing time compared to both C and MATLAB implementations. Wachowiak et al. [16] proposed an asynchronous GPU-based approach of PSO. Each particle is handled by a separate GPU thread, where they run a specified number of iterations after which they are resynchronized.

Mussi et al. [17] also present an asynchronous GPU implementation of the PSO algorithm. The authors aspire to overcome the drawbacks of asynchronous PSO imposed by the master-slave approach. In their method, the neighborhood is updated immediately after a particle is evaluated. The same authors compared their asynchronous implementation against their synchronous GPU version of PSO where they noticed speedups ranging from 5 to 35 times, depending on the problem’s dimensions [27].

Hung et al. [18] propose a synchronous GPU implementation of the PSO method, meaning that the entire process is executed on GPU. Updating velocity, position, evaluating the given function, and updating best local and global candidate solutions are all implemented as separate CUDA kernels. This implies that, although the implementation is sped up by use of a GPU, the method is still synchronous.

Wende et al. [28, 29] observed that the GPU can handle large amounts of work, but that small-scale workloads are expensive to evaluate on a GPU. When doing so, the cost of shipping work towards and from the GPU is rather high. Using the Hyper-Q feature of NVIDIA’s GPUs, the authors provided a single, shared, GPU of work using multiple CPU threads. This feat can assure that the resources of both the CPU and the GPU are efficiently used.

## Solving epidemiological models on GPUs

This paper proposes that infectious disease dynamic models are evaluated in parallel on the GPU. Since the interest lies with inferring parameters of infectious disease models, the following section outlines the characteristics of said models. Second, a comparison is made between the proposed approach and the previously mentioned ones, showing where performance is gained. Third, both the integration method and the right-hand of the ODEs can benefit from parallelization as vector operations and matrix-vector multiplications (i.e. calculating the force of infection) can easily be performed on GPU.

### The SIR model

A basic model that is often used to study infectious disease dynamics in a population, is the Susceptible-Infected-Recovered or SIR model which describes the flow of individuals through these mutually exclusive disease states. In terms of the SIR model, the following set of partial differential equations (PDEs) can be used to model these dynamics,

1 $$ \left\{ \begin{array}{lll} \frac{\partial S(a,t)}{\partial a}+\frac{\partial S(a,t)}{\partial t}&=&-(\lambda(a,t)+\mu(a))S(a,t),\\[1ex] \frac{\partial I(a,t)}{\partial a}+\frac{\partial I(a,t)}{\partial t}&=&\lambda(a,t)S(a,t)-(\nu+\mu(a))I(a,t),\\[1ex] \frac{\partial R(a,t)}{\partial a}+\frac{\partial R(a,t)}{\partial t}&=&\nu I(a,t)-\mu(a)R(a,t). \end{array}\right.  $$

where *S*(*a*,*t*), *I*(*a*,*t*), and *R*(*a*,*t*) are the age- and time-specific (*a* and *t*) number of susceptibles, infected and recovered, respectively, with *S*(0,*t*)=*B*(*t*) the number of newborns at time *t*. In this paper, the basic SIR model is used, whereas in practice this model is commonly extended (e.g. Goeyvaerts et al. [30] use a SEIRS model). Such extensions typically exhibit the same dynamics and characteristics as the basic SIR model.

The force of infection *λ*(*a*,*t*) is given by the mass action principle:

$$\lambda(a,t)=\int_{0}^{+\infty}\beta(a,a^{\prime},t)I(a^{\prime},t)da^{\prime}. $$

where *β*(*a*,*a*^′^,*t*)≡*β*(*a*,*a*^′^) is often assumed to be time-independent and governed by so-called Who Acquires Infection From Whom (WAIFW) matrices, i.e. mathematically convenient structures or by the social contact hypothesis (see e.g. [31]).

**Fig. 1:**
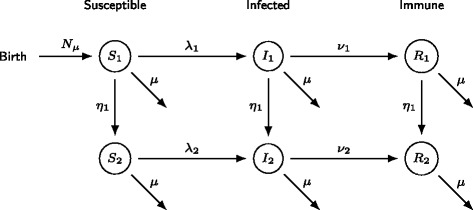
Flow diagram for the CAS implementation of the SIR model. The individuals enter the susceptible class, then move to the infected class (at rate *λ*) and after recovering they move into the immune class (at rate *v*). *η*_*i*_ is the transition rate from category *a*_*i*_ to *a*_*i*+1_, and *μ* is the mortality rate in this diagram

Solving a set of PDEs is not straightforward and depends on specific assumptions made for the different model parameters. In practice most PDE models are approximated using different methods in order to reduce the PDE model to a more solvable and workable model. Two well-known procedures are often used in mathematical epidemiology [32]: (1) The Cohort Age Structured (CAS) model replaces the set of PDEs with a set of ordinary differential equations (ODEs) by considering *K* compartmental models representing *K* age-groups and using continuous transitions from one age-group to the next (see Fig. 1; *η*_1_). (2) In a Realistic Age Structured (RAS) model individuals change status from S to I and from I to R during one year (assuming age-groups of one year) after which they instantaneously move to the next age-group (see e.g. [30]). It is often said that a RAS-model better reflects infectious disease dynamics because children switch grades in school generally at the same moment during the year, and only once per year (see e.g. [33]). For a more in depth discussion about the advantages and disadvantages of the different methods to discretize age-structured PDE models, the reader is referred to [32, 34, 35].

This paper focusses mostly on the CAS model, but the RAS model would be fairly similar. The CAS model uses a backward in space finite difference scheme [36], comparable to the method of characteristics [37], essentially breaking down the PDE into the following set of coupled ODEs:

2 $$ \left\{\begin{array}{lll} \frac{S(a, t) - S(a - \Delta a, t)}{\Delta a}+\frac{\partial S(a,t)}{\partial t}&\,=\,&-(\lambda(a,t)+\mu(a))S(a,t),\\ \frac{I(a, t) - I(a - \Delta a, t)}{\Delta a}\,+\,\frac{\partial I(a,t)}{\partial t}&\,=\,&\!\lambda\!(a,t)S(a,\!t\!)\,-\,(\nu\!\,+\,\mu(a))I\!(a,\!t)\!,\\ \frac{R(a, t) - R(a - \Delta a, t)}{\Delta a}+\frac{\partial R(a,t)}{\partial t}&\,=\,&\nu I(a,t)-\mu(a)R(a,t). \end{array}\right.  $$

Note that using this discretization, the force of infection can be rewritten as:

3 $$ \lambda(a,t)=\sum\limits_{a^{\prime}} \beta(a,a^{\prime}) I(a^{\prime},t).  $$

To ensure stability of the scheme, a necessary and sufficient condition is the Courant-Friedrichs-Lewy condition [36] which requires *Δt*≤*Δa*.

### Runge-Kutta-Fehlberg on GPU

With many methods to choose from, the Runge-Kutta-Fehlberg method, commonly known as *RK45*, is characterized as being an integration method that provides the most bang for the buck [20]. In his paper [38], Fehlberg describes a fifth-order method with six function evaluations where another combination of the same six functions gives a fourth-order method. The difference between these estimates approximate the truncation error, which in turn adjusts the step size. Related work shows that there have been successful attempts at porting a Runge-Kutta method to GPU. However, in most of those implementations, each GPU thread evaluates a system of differential equations of varying task-length, due to analyzing different parameter candidates for inference and given the SIMT nature of GPU, it is difficult to minimize performance loss. The following sections will outline our approach of implementing an efficient *RK45* implementation on GPU.

#### Using a block of threads

The proposed method differs from existing techniques in that a block of threads, mapped on a single streaming multiprocessor (SM), jointly integrate a single ODE while different blocks are working on other ODEs. This has the advantage that threads can communicate via shared memory instead of global memory (approximately 60x faster) [39]. However this technique only works for small systems that fit in shared memory. The reduction step in the *RK45* method would normally require a synchronization barrier, but can be avoided when using a block of threads through the use of warp shuffle functionality of CUDA [39]. As a result the threads can execute the *RK45* method in lock-step.

#### Calculating the force of infection

**Fig. 2:**
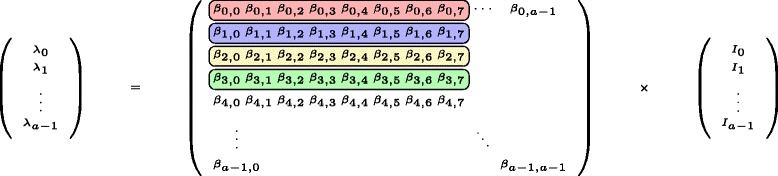
Being the most computational intensive part of the evaluation, all the threads are divided up to rapidly compute the FOI, *λ*. For illustrative purposes, there are 32 threads (in total) active in this example. There are four lambdas calculated in parallel; for each lambda there are eight threads in charge of executing the actual matrix-vector calculation. This window of eight threads per row slides over the elements of the row until the lambda is completely calculated

Another integral part of the integration process is the right-hand side of the ODE, which can also benefit from parallelization. Multiplying the social contact matrix *β* with a vector of infected *I*, results in the force of infection (FOI) *λ*, as depicted in Fig. 2 and Eq. 3. Given that at each iteration of the integration process *I* is updated based on an update of *λ*, and this multiplication must be performed several times, it is the most computational intensive part of the integration process.

Figure 2 shows that available threads are divided up in order to calculate the FOI. The division scheme, describing how many threads are used per row, is chosen based on the GPU architecture. When calculating a single *λ* value, the warp shuffle feature of CUDA is used to perform reductions.

## Asynchronous parameter inference

The next, and final, step is to infer model parameters so that the SIR model accurately describes the observed data by maximizing the (log)likelihood of the model, also called maximum likelihood estimation (MLE). This entails that the model is integrated using candidate parameters, applying the ODE integration technique introduced in previous sections. This paper proposes the use of an asynchronous particle swarm method for parameter inference with the goal of fully utilizing both CPU and GPU.

### Particle swarm optimization

Particle swarm optimization, introduced by Kennedy et al. in 1995 [13], is based on the principles of bird-flocking, fish schooling, and swarm theory. Individual members of the society can profit from the discoveries made by other members during the search for food. The same principle is applied to explore the parameter space of (non-linear) problems.

Particles have both a position and velocity which are updated at every iteration using the particles’ best known local (*P*_*best*_) and global position (*g*_*best*_). The vanilla method decomposes into two steps, as described in Algorithm 1, namely to first initialize all particles and then update the particles’ position and velocity until a termination criterion has been met.

Each particle represents candidate parameters for the SIR model and will be evaluated by first integrating the ODE using the proposed GPU implementation of the *RK45* method and then passing the result to the (log)likelihood function. While the evaluation can be performed in parallel, alternating between GPU and CPU results in inefficient resource utilization. Only one processor is making progress at any given time. In addition, if some particles take longer to evaluate, resource utilization drops further.



### Asynchronous particle swarm optimization

**Fig. 3:**
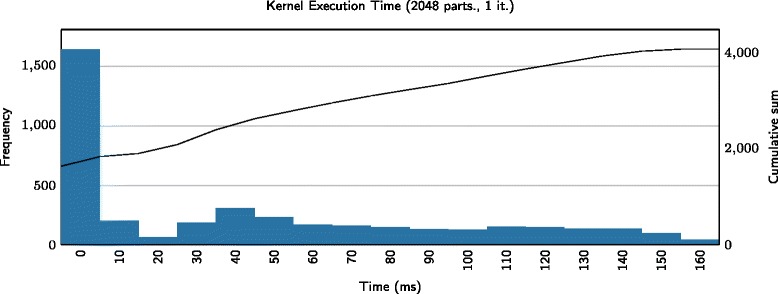
Evaluating the model with different candidate parameters together with different execution timings. This figure shows the result of employing the PSO algorithm with 2048 particles and with one iteration step. The x-axis depicts the execution time in milliseconds and y-axis depicts the number of particles that have been executed within the amount of time given by the x-axis. With only one iteration, particles are initialized all over the parameter space

The vanilla PSO algorithm (Algorithm 1) requires that all particles have updated their velocity and position before advancing to the next iteration. On the other hand, asynchronous PSO [25, 26, 40] removes this barrier with the goal of keeping all processing units from idling. The key to implement an asynchronous PSO algorithm is to separate the update actions for individual particles from those associated with the swarm as a whole. While the asynchronous algorithm might require additional iterations due to some particles running ahead of others, the convergence rate is generally reported as comparable and the algorithm significantly outperforms its synchronous counterpart in terms of parallel efficiency [25, 26]. This is especially true when the evaluation time of a particle depends on its position, which is the case for numerical integration of ODEs (as also noted by Murray [24]). Figure 3 shows a histogram of the execution times for the evaluations of 2048 particles in the first iteration of the PSO algorithm. As the execution time varies between 0ms and 160ms, the synchronous algorithm will have significant idling of processing units. In the asynchronous algorithm, faster particles can advance to the next iteration and will positively influence the slower ones, as information is shared between the whole population of particles.

### Heterogeneous approach

The key idea to fully utilize both CPU and GPU resources is to produce many concurrent GPU workloads which as a whole can fill up the GPU to capacity and asynchronously offload them such that the CPU can concurrently pre/post-process the workloads. The Concurrent Kernel Execution (CKE) and Hyper-Q features introduced by NVIDIA with the Fermi and Kepler architectures, respectively, enables this by allowing multiple CPU cores to simultaneously launch concurrent workloads on a single, shared, GPU. The Hyper-Q feature allows 32 simultaneous, hardware-managed connections (or work queues), compared to the single work queue available on the Fermi architecture.

When dealing with imbalanced workloads, it is advantageous to have multiple kernels executing concurrently instead of a single monolithic kernel as the latter would idle GPU resources while finishing the longest of tasks. CKE on the other hand allows the GPU to continue execution with the next workloads. Since offloading to GPU can be costly due to data transfer, kernel launching and synchronization [14], particles are grouped into batches to amortize these costs. Given that execution time of a batch is determined by the slowest particle in the batch, there is a trade-off between batching and the ability to deal with the imbalance.

It is important to note that, while Hyper-Q allows offloading of multiple kernels, the newer generation of GPUs still only have two copy engines. This implies that only two data transfer operations can happen simultaneously: one transfer from device to host memory and one in the opposite direction. This could result in a transfer bottleneck with a drop in performance as a result.

The CPU implementation of the particle swarm algorithm has two goals: maximize CPU utilization and separate the optimization algorithm from GPU details. Ideally, the optimization algorithm should be completely oblivious to anything GPU related such that it is easy to reuse existing implementations and switch between different algorithms.

**Fig. 4:**
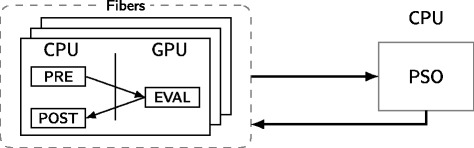
The PSO algorithm is run on CPU side, while the evaluation of the particles is handled by the GPU. CPU cores send batches of particles to the GPU asynchronously in order to increase performance. Before a batch is sent to the GPU, some pre-processing can be performed, as well as eventual post-processing when the evaluation is finished

The former is accomplished by multiple CPU threads processing particles in parallel thus utilizing all cores on the host and through the use of fibers (or coroutines [41]). Fibers are light-weight threads of execution that enable cooperative multitasking. This means that a fiber must yield its execution to enable the execution of another. In the proposed method, each CPU thread handles one or more fibers (see Fig. 4). Whenever the thread must wait for the GPU to finish, it switches to another fiber and continues processing different particles: either pre-processing, offloading to GPU or computing the likelihood. The use of fibers also enables a single CPU core to process multiple particles at the same time and to occupy the 32 Hyper-Q connections.

While the use of fibers is not necessarily required to maximize CPU usage, it makes the implementation of the PSO algorithm much easier and free of any GPU related details. The alternative would be to manage the Hyper-Q work queues manually inside the algorithm. The proposed implementation consolidates such details in the GPU implementation.

For every Hyper-Q work queue, the implementation also keeps several work packages containing CPU pinned memory and GPU device memory such that it can avoid expensive memory allocation for every offload. These packages are placed in a concurrent first-in first-out data structure from which the CPU threads can acquire them whenever an evaluation is offloaded. In this case, the concurrent_queue implementation of the TBB template library [42], developed by Intel, is used.

## Results and discussion

In this section, the performance and behavior of the *RK45* implementation as well as the asynchronous parameter inference using a heterogeneous architecture is described. The experiments were conducted on a machine with four eight-core Intel Westmere processors running at 2.67GHz and an NVIDIA GeForce GTX TITAN X. The machine runs the GNU/Linux operating system and CUDA 8.0 was used for programming the GPU.

### Scalability and performance of *RK45* on GPU

**Fig. 5:**
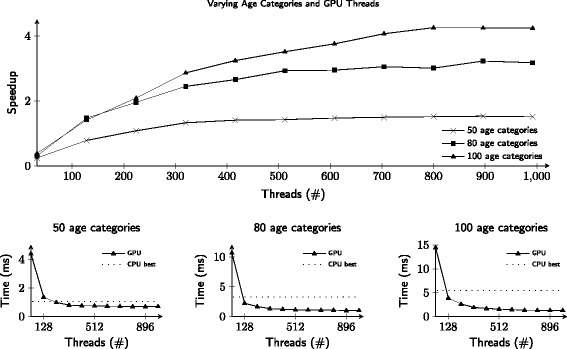
The bottom row depicts the model execution timings on GPU when using three different amounts of age categories. The x-axis depicts the amount of GPU threads used when evaluating an ODE, while the y-axis depicts the execution timing in milliseconds. The number of operations is in the order of $\mathcal {O}(n^{2})$, meaning that when comparing 50 and 100 age categories, the latter has 4 times as many operations. One can see that when there are more age categories available, the better the GPU will perform when compared to the CPU. The top row depicts the speedup of the GPU when compared with the best CPU timing for each of the three age category settings

Figure 5 shows the speedup in terms of number of GPU threads used of the *RK45* implementation when integrating a single ODE as well as a performance improvement of between 1.5 and 4.23 times compared to a CPU implementation. The number of operations of this model is in the order of $\mathcal {O}(n^{2})$, meaning that when comparing 50 and 100 age categories, the latter has 4 times as many operations. As noted by previous work [21–23], small-scale ODE integration cannot fully utilize the GPU. By increasing the amount of age categories, the number of ODE equations grows proportionally and allows more GPU threads to be effectively used.

**Fig. 6:**
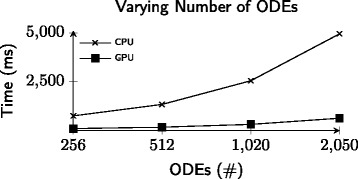
Each line represents the execution time for CPU and GPU when integrating an increasing number of ODEs. The x-axis depicts the number of ODEs used and the y-axis depicts the execution time in milliseconds. Both approaches scale linearly when given more work to process with the GPU remaining competitively faster

Filling the GPU up to capacity, however, requires an even larger ODE system. In contrast, the approach taken by this paper is to integrate many small-scale ODEs at the same time. Figure 6 shows that the execution time grows linearly for both CPU and GPU as the number of ODEs increases. Thus the GPU implementation remains up to 4 times faster, regardless of the amount of work.

The following experiments always use the introduced SIR model with 100 age-categories as it is commonly used and represents a realistic range of ages.

### Particle swarm optimization

To demonstrate the performance of the heterogeneous approach compared to the CPU-only implementation, both synchronous and asynchronous versions of the PSO algorithm are run with 256 and 2048 particles. Execution is terminated after 100 iterations, under the assumption that convergence rates are the same for both versions, in order to effectively compare execution times. All tunable parameters, such as number of CPU/GPU threads and fibers, are explored and the best configuration is reported.

**Fig. 7:**
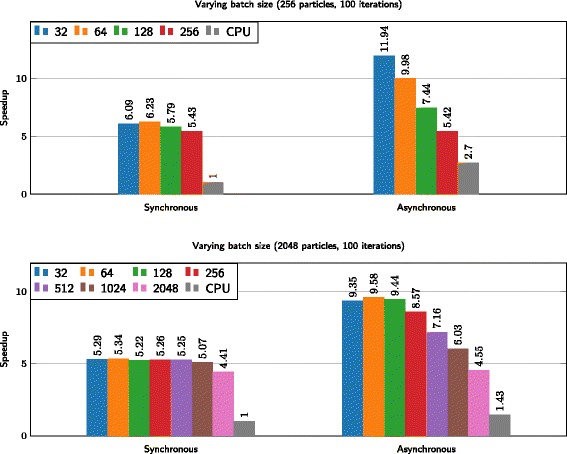
Employing the heterogeneous, asynchronous, PSO algorithm using 256 and 2048 particles, respectively. Each bar represents the execution time in milliseconds and each color represents a different batch size. The figure depicts that the asynchronous approach not only benefits from a fast integration method on GPU, but keeping both CPU and GPU busy with useful work is more preferable when compared to the synchronous approach. Given possible imbalance when evaluating ODEs, it is advisable to evaluate the particles in small batches

Results are illustrated in Fig. 7. The synchronous version benefits from the acceleration of the ODE integration on GPU. However, as shown in Fig. 3, integration times can vary significantly for different candidate values. The trade-off in terms of batch size is clearly visible, even in the synchronous case. Decreasing the batch size allows CPU threads to commence post-processing more quickly, instead of waiting for the slowest integration to finish. As the barrier prohibits fast batches of particles to continue to the next iteration, this benefit is not substantial. The maximal speedup measured was 6.2 and 5.3 when using 256 and 2048 particles, respectively.

The asynchronous implementation removes the latter problem, permitting some particles to run ahead of slow particles, resulting in a significant speedup and the batch size playing a more important role. The trade-off between the ability to deal with imbalance and the overhead of offloading multiple batches is clearly visible; a batch size that is too small introduces too much overhead. Maximal speedup measured for the asynchronous implementation was 12 and 10 when using 256 and 2048 particles, respectively.

**Fig. 8:**
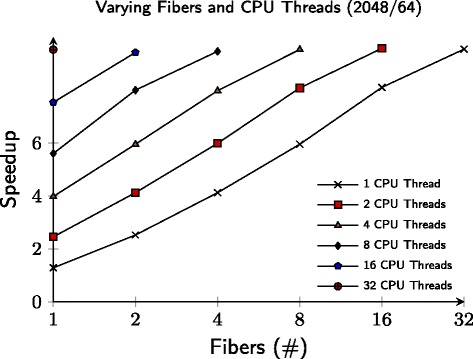
Given that batches of particles can be evaluated asynchronously, one can ship off multiple batches of particles to the GPU in parallel. Once a CPU thread ships off a batch of particles to the GPU, it needs to wait for it to be finished before continuing to work. Using fibers, a CPU thread can process multiple batches at the same time and therefore increases performance. The x-axis depicts the amount of fibers per CPU thread and the y-axis depicts the speedup of the GPU compared to the best CPU timing. A total of 2048 particles were used with 64 particles per batch

Figure 8 shows that the use of fibers has no significant influence on execution time. For example, 32 CPU threads, each one fiber, performs equally well when compared to using one CPU thread with 32 fibers. The same can be said over a moderate, eight-core system with four fibers.

## Conclusion

A crucial part in performing parameter inference for epidemiological models is integrating a set of ODEs. The efficient implementation of *RK45* on GPU is capable of integrating up to 4 times faster than a CPU implementation. Concurrent offloading of many small-scale ODEs can fill up the GPU to capacity while maintaining this performance. The efficiency of the implemented *RK45* method can also be improved by making it customized to the SIR model instead of the generic approach proposed in this paper.

The proposed method keeps optimization methods on the CPU. Such a heterogeneous approach to asynchronous particle swarm optimization, as described in this paper, provides a way to perform parameter inference while efficiently utilizing resources of both CPU and GPU and decreases execution time. Evaluating all particles at once, the overall execution time is determined by the slowest evaluation. Grouping particles into batches mitigates this variance in execution time and increases performance. The removal of the synchronization barrier, prohibiting particles to continue to the next iteration, allows certain batches to run ahead of other batches. Note, however, that a trade-off has to be made between the ability to deal with imbalance and the overhead of offloading multiple batches of particles. The optimal batch size in our experiments achieves a speedup up to 12 times.

The proposed contributions enable researchers, for example, to deal with urgent situations like epidemic outbreaks, where a rapid assessment of intervention strategies is required. Future research should ascertain how these contributions perform on newer GPU architectures as well as how they perform on real-life epidemiological scenarios. Also, the proposed approach has been programmed to work with all available GPUs on a host system. However, such a setup has not been evaluated by the authors and is well worth looking into.

## Abbreviations

CAS: Cohort age structured
CPU: Central processing unit
CKE: Concurrent kernel execution
FOI: Force of infection
GPU: Graphics processing unit
ODE: Ordinary differential equation
PSO: Particle swarm optimization
PDE: Partial differential equation
RAS: Realistic age structured
RK45: Runge-Kutta-Fehlberg
SIR: Susceptible-infected-recovered
SIMD: Single-instruction multiple-data
SIMT: Single-instruction multiple-threads
WAIFW: Who aquires infection from whom
SM: Streaming multiprocessor
MLE: Maximum likelihood estimation
